# Analysis of CT and MRI Manifestations of Joubert Syndrome

**DOI:** 10.5334/jbsr.3283

**Published:** 2023-09-29

**Authors:** Da-wei Liao, Xue Zheng

**Affiliations:** 1 Department of Radiology, the Affiliated Hospital of Southwest Medical University, Luzhou, China

**Keywords:** Joubert syndrome, MRI, CT, dysplasia of the cerebellar vermis

## Abstract

**Objective:**

To investigate the computed tomography and magnetic resonance imaging manifestations of Joubert syndrome (JS).

**Method:**

In this retrospective analysis, we investigated the clinical and imaging characteristics of JS in a cohort of twelve pediatric patients with confirmed diagnoses. Specifically, we analyzed both computed tomography (CT) and magnetic resonance imaging (MRI) manifestations in this population. CTs were performed on four patients and MRIs were performed on twelve, respectively.

**Results:**

JS is characterized by specific CT and MRI findings, including midline fissure, batwing, or triangular formations of the fourth ventricle between the bilateral cerebellar hemispheres, and molar sign at the midbrain level. All twelve cases in this cohort exhibited these traits, along with other cerebral abnormalities, such as dysplasia of the corpus callosum in two cases, gray matter heterotopia in one case, and occipital meningocele in one case.

**Conclusion:**

JS has distinctive CT and MRI characteristics that can be clinically identified.

## Introduction

Joubert syndrome (JS) is a rare congenital cerebral developmental malformation initially described by Joubert in 1969 [1]. A similar occurrence was reported by Boltshauser in 1977 [2], which is also called Joubert-Boltshauser syndrome. JS exhibits certain clinical features including hypotonia, ataxia, mental retardation, developmental delay, respiratory dyskinesia, facial abnormalities, and specific eye movements. JS can affect multiple organs, including the liver, kidney, retina, and bones [3]. We conducted a retrospective analysis of the characteristics of twelve cases to improve understanding and diagnosis of JS.

## 1 Materials and Methods

### 1.1 Clinical data

We retrospectively collected clinical information on cases of JS diagnosed between January 2010 and December 2022 using our hospital’s medical record system. A total of 12 pediatric cases were included in the analysis, with a gender distribution of seven males and five females. The age range of the patients varied from two months to 12 years, with a median age of 4.5 years. All 12 pediatric patients exhibited varying degrees of developmental delay, mental retardation, and hypotonia. Of these, eight displayed abnormal eye movements, including four cases of nystagmus, two cases of strabismus, and two cases of amblyopia. In addition, nine patients had respiratory abnormalities, characterized by apnea or shortness of breath during the neonatal period, and six had epilepsy. The children’s families were fully informed of the study and provided informed consent, and the study was approved by the hospital’s ethics committee.

### 1.2 Imaging methods

All 12 children underwent MRI examinations, and four of them underwent simultaneous CT examinations. The parameters used for the MRI and CT scans were as follows.

MRI examination was carried out using the PHILIPS Achieva 3.0T MR apparatus for routine axial, sagittal, and coronal scanning. T1WI and T2WI are Turbo Spin Echo sequences. T1WI (TR 488 ms, TE 15 ms) and T2WI (TR 37511ms, TE 100ms). The slice thickness was 5.5 mm with a slice distance of 1 mm. The flip angle (FA) used was 90°.

CT examination was carried out using the PHILIPS 256-slice Brilliance iCT scanner. The voltage used was 120 KV, and the tube current was adjusted to 240 mAs. The scanning matrix was 512 × 512, and the field of view was 25 × 25 cm. The slice thickness was 5 mm, and the pitch ratio was 0.392. All scans were performed with the patient in the supine position, and the auditory canthus line (OM line) applied as the baseline.

If necessary, the children were sedated with oral administration of 10% choral hydrate, 30 minutes prior to the examination.

### 1.3 Diagnostic criteria

In 1992, Saraiva and Baraistr introduced a set of five diagnostic criteria for this disorder [4]: (1) hypoplasia or absence of cerebellar vermis development; (2) hypotonia during infancy; (3) intellectual disability; (4) abnormal breathing patterns (including episodic apnea and dyspnea); and (5) oculomotor abnormalities (including nystagmus and strabismus). A patient who meets all of criteria 1–3 and at least one of criteria 4–5, reaches conditions for this diagnosis.

## 2 Results

**Figure 1:**
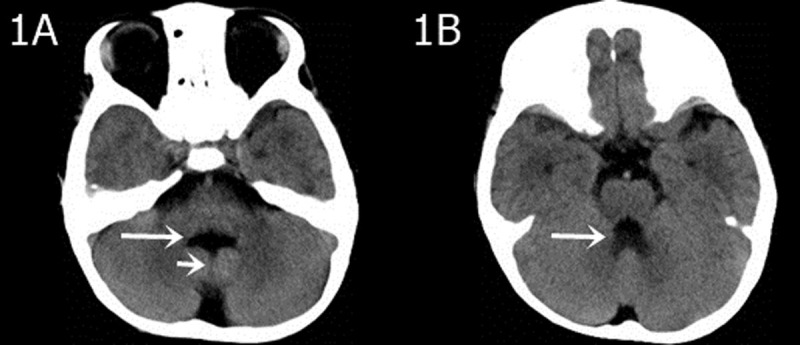
A three-year-old boy with a speech disorder and lifelong difficulty understanding simple instructions. **A)** A hypoplastic cerebellar vermis is evident on the axial CT image, with a midline fissure sign between the bilateral cerebellar hemispheres (short arrow), and a batwing sign on the fourth ventricle (long arrow). **B)** The upper cerebellar peduncles are thickened and elongated, forming a molar sign (arrow) with the midbrain.

**Figure 2:**
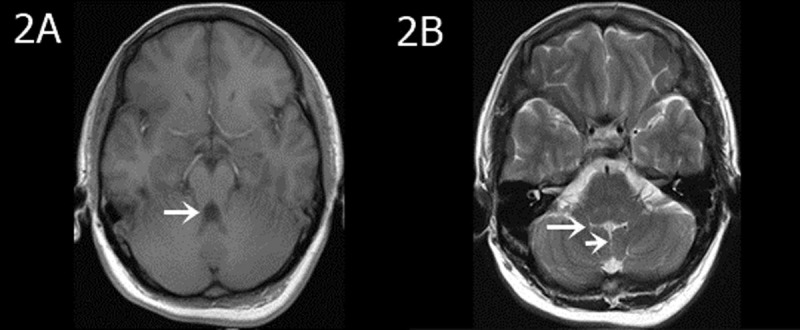
An eight-year-old girl with a three-year developmental delay. Fine motor skills of the hands were deficient, and separation movements were insufficient. **A)** The T1WI transverse view revealed a molar sign (arrow). **B)** The T2WI transverse view showed bilateral interhemispheric midline fissure sign (short arrow) and fourth ventricle batwing sign (long arrow).

**Figure 3:**
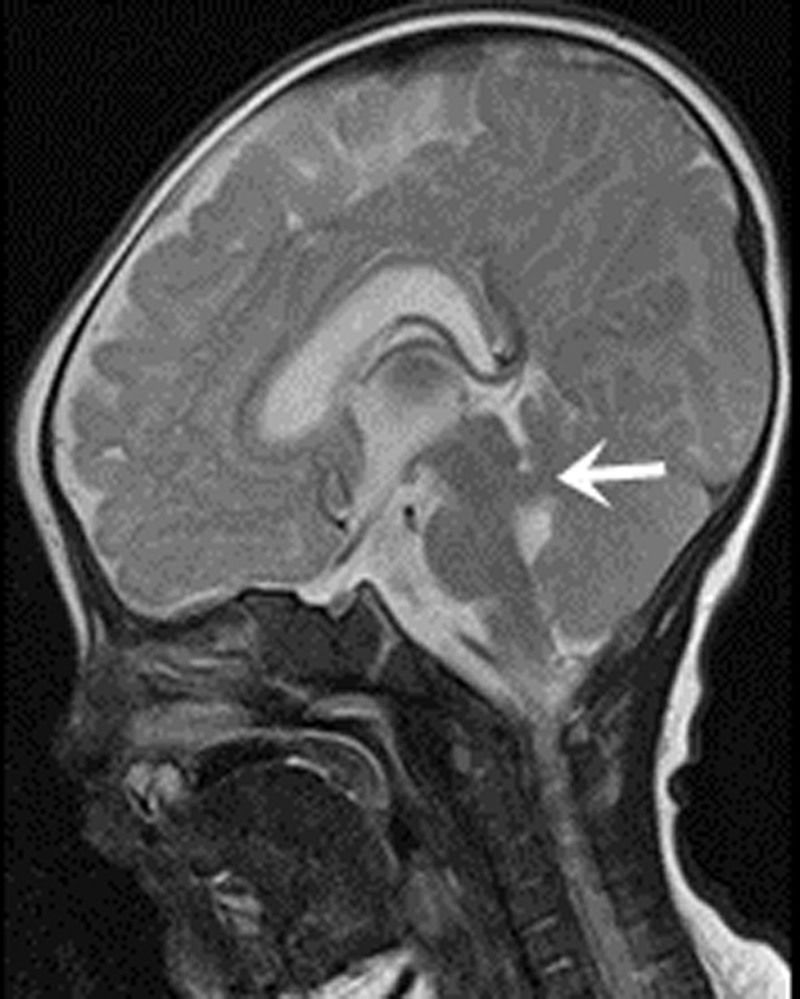
A male infant, aged two months, presented with hoarseness for one day and head shaking for half a day. The sagittal T2WI revealed slight thinning of the midbrain, slight enlargement of the interpeduncular fossa, and thickening and elongation of the upper cerebellar peduncle, which was running horizontally (indicated by arrow).

**Figure 4:**
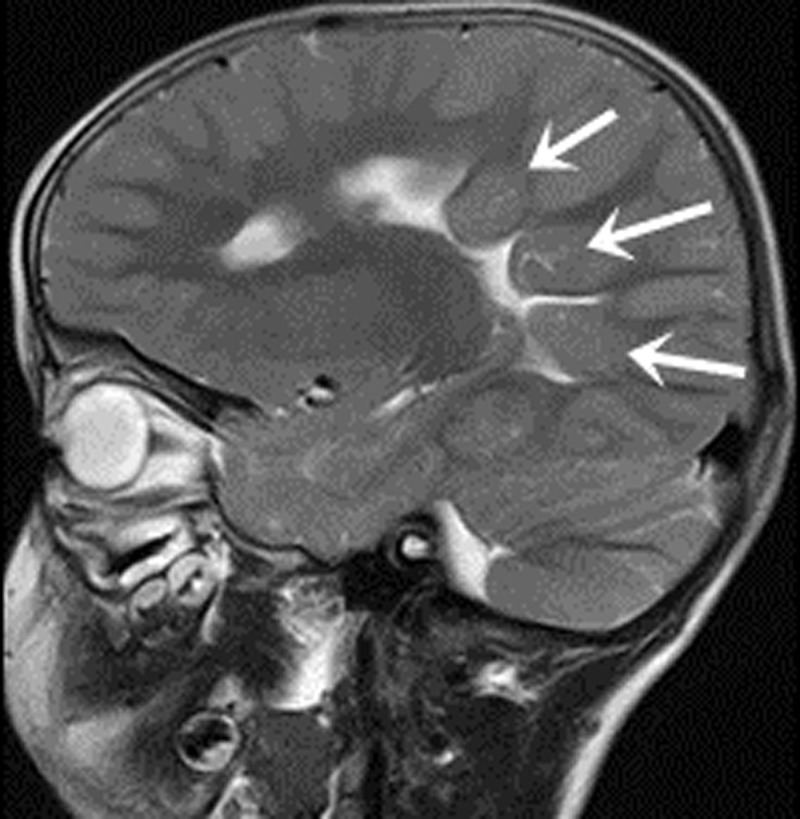
A three-year-old male toddler with difficulty walking unassisted, poor verbal skills, and developmental delays. A sagittal T2WI scan revealed the absence of the body and splenium of the corpus callosum, and the gray matter heterotopia (arrows).

**Figure 5:**
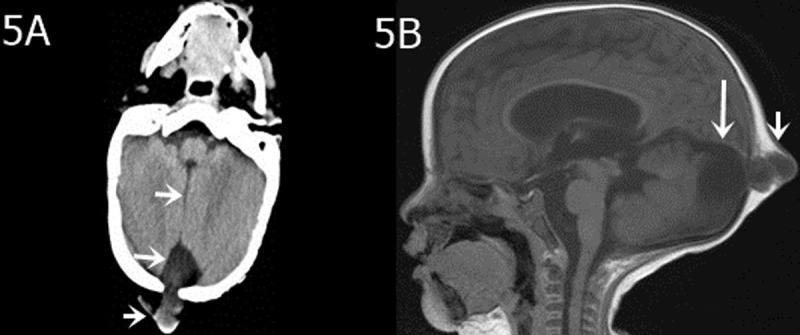
A seven-month-old girl with a pulsating occipital mass lasting seven months. **A)** The midline fissure (arrow) between the bilateral cerebellar hemispheres, the great occipital fossa (long arrow), and a localized occipital bone defect with accompanying meningeal and brain herniation (short arrow). **B)** Enlargement of the posterior fossa cistern (long arrow) and localized bony defect with accompanying meningeal herniation (short arrow) are visible in the sagittal T1-weighted image.

In all 12 cases, patients presented with the following characteristics: midline fissure (Figures 1A, 2B and 5A), and a batwing-shaped or triangular fourth ventricle (Figures 1A and 2B), molar tooth sign (Figures 1B and 2A), and thickening of the horizontal aspect of the cerebellar vermis (Figure 3). The following associated intracranial abnormalities were also observed: corpus callosum dysplasia in two cases (Figure 4), gray matter heterotopia in one case (Figure 4), and occipital meningocele and enlarged cisterna magna in one case (Figure 5).

## 3 Discussion

### 3.1 Pathogenesis and pathological features

JS is a congenital dysplasia impacting the midbrain and rhombencephalon. It is an autosomal recessive genetic disorder with an incidence of 1 per 100.000. [4]. It predominates in male children, with a male-to-female ratio of 3:2 [5]. As in previous reports, our cohort showed a high proportion of male children individuals.

It is associated with over 20 gene loci, the chief ones being located on chromosomes 9q34.3, 11p12-q13.3, and 6q23. The pathogenic gene encodes primary cilia and abnormalities in the coding of appendage proteins. These abnormalities impair the formation of the left and right axes of the embryo, limb development, and neurogenesis [6]. Out of the cases in this group, three possessed a family history of the disease, while one had parents who were cousins and had a consanguineous marriage.

In 2012, Paprocka et al., divided Joubert syndrome into four phenotypes based on liver, kidney, and eye involvement [7]. These were as follows: (1) simple Joubert syndrome; (2) Joubert syndrome with eye defects; (3) Joubert syndrome with liver and kidney involvement; and (4) Joubert syndrome with orofacial and digital defects. All 12 cases in this study belong to the simple type of Joubert syndrome. No combined liver, kidney, eye, or other defects were detected.

The neuropathological alterations observed in JS encompass the absence or underdevelopment of the cerebellar vermis, deformation of the dentate nucleus, displacement or underdevelopment of the pontine basal ganglia and medullary nerve nuclei, and nearly complete absence of the corticospinal tracts. Other anomalies, such as facial dysmorphism, microcephaly, superficial occipital meningeal fissures, gray matter anomalies, corpus callosum dysplasia, and congenital heart disease, are also found in some cases [8]. The cases studied here all featured either absence or dysplasia of the cerebellar vermis, with one case manifesting additional dysplasia of the corpus callosum and gray matter heterotopia, and another displaying occipital meningoencephalocele. Characteristic symptoms of JS mainly include hypotonia and ataxia, though they also include developmental delays, cognitive deficits, and abnormal breathing patterns [9]. All cases in this group exhibited developmental delay, as well as hypotonia and hyperventilation with gasping in infancy, consistent with previous studies.

### 3.2 CT and MRI manifestations

In our study, highly noticeable CT and MRI imaging features of JS encompass the molar tooth sign, as well as the midline fissure, and a batwing-shaped or triangular fourth ventricle. Corpus callosum dysgenesis, septal defects, gray matter displacement, hippocampal malrotation, posterior fossa meningeal herniation, and ventricular dilatation are also often observed.

The “molar tooth sign” may be identified through various characteristics, such as the widening of the midbrain, deepening of the interpeduncular cistern, thickening and parallel orientation of the superior cerebellar peduncle, and a distinct “molar shape” in the nearby cerebrospinal fluid. These features are readily visible in transverse CT and MRI scans. The molar tooth sign in JS patients is attributed to the abnormal intersection of the pyramidal tract and the superior cerebellar peduncle fiber bundles. This causes thickening and elongation of the superior cerebellar peduncle, which runs vertically between the midbrain and cerebellum. A lack of fibers in the superior cerebellar peduncle causes shortening of the anterior–posterior diameter of the midbrain, creating a deeper interpeduncular fossa [4]. Some experts believe molar tooth sign on images to be a clear diagnostic indicator for the disease. However, it is essential to note that not all cases will display this particular imaging feature [10].

The abnormal development of the cerebellar vermis can lead to the formation of distinct morphological features in the fourth ventricle, such as the midline fissure, batwing, or triangular shape. The term “midline fissure” here refers to the absence of a normal cerebellar vermis with a molar shape where the bilateral cerebellar hemispheres are not connected, but rather situated closely together. In this condition, cerebrospinal fluid fills a slender, continuous pathway that communicates with the fourth ventricle, giving rise to a distinct midline fissure appearance on axial images. The fourth ventricle presents a distinctive form, characterized by a batwing-like upper portion and a triangular middle portion caused by the abnormal development of the cerebellar vermis.

Each case in this group showed a molar tooth sign, midline fissure, batwing, or triangular fourth ventricle in their CT or MRI scans. These cases also presented other abnormalities: one case exhibited corpus callosum dysgenesis and gray matter heterotopia, and another displayed occipital meningoencephalocele. Although specific imaging manifestations such as the molar shape, midline fissure, batwing, or triangular fourth ventricle are often associated with JS, it is crucial to understand that not all patients with the syndrome show these characteristics. Furthermore, these imaging manifestations are not exclusive to JS and may appear in other conditions [11].

### 3.3 Advantages of MRI in imaging of JS

CT axial imaging can show the molar tooth sign, batwing, or triangular fourth ventricle clearly. However, due to interference from posterior fossa bone artifacts, the midline fissure may not be readily visible which can lead to misdiagnosis. Visualizing the thickened superior cerebellar peduncle requires CT post-processing reconstruction.

MRI outperforms CT in showing brainstem structures because of superior spatial resolution and soft tissue contrast. Specifically, MRI can clearly show the thin line of cerebrospinal fluid signal between bilateral cerebellar hemispheres and the fourth ventricle for the midline fissure. With its ability to generate multi-plane and multi-directional images, MRI can show the cerebellar vermis and corpus callosum anatomy in the sagittal plane clearly. MRI can reveal the complete or partial absence of the cerebellar vermis and the development of the corpus callosum, including any defects or poor development in the body and tail. The sagittal plane of MRI offers clearer visualization of the thickened and parallel superior cerebellar peduncles. MRI also outperforms CT in detecting gray matter shifts. In clinical practice, MRI is the preferred imaging modality for diagnostic purposes when the clinical symptoms align with JS. In the cases studied, both CT and MRI scans revealed characteristic imaging features of JS.

## 4 Differential Diagnosis

JS needs to be differentiated from the following conditions: (1) Dandy-Walker syndrome. This is distinguished by the absence of the cerebellar vermis, posterior cranial fossa enlargement filled with cerebrospinal fluid-containing cysts, posterior and superior expansion of the fourth ventricle, and separation and anterior and lateral retraction of bilateral cerebellar hemispheres. In Dandy-Walker syndrome, brainstem development is normal, and the molar tooth sign is absent. (2) Rhombencephalosynapsis, characterized by the absence of the cerebellar vermis and fusion of bilateral cerebellar hemispheres without visible separation, often called the “cleft sign”. (3) Down syndrome can be differentiated from JS by genetic diagnosis (karyotype trisomy 21).

## 5 Conclusion

In conclusion, the main pathological changes inherent in JS are located in the vermis of the cerebellum and are characterized by distinct imaging features. These include a midline cleft between the bilateral cerebellar hemispheres, a batwing, or triangular fourth ventricle, and the presence of molar tooth sign. Other abnormal manifestations, such as corpus callosum dysgenesis and gray matter heterotopia, may be observed. By combining these clinical manifestations, a definitive diagnosis can be established.
